# The University Medicine Greifswald’s Trusted Third Party Dispatcher: State-of-the-Art Perspective Into Comprehensive Architectures and Complex Research Workflows

**DOI:** 10.2196/65784

**Published:** 2024-11-29

**Authors:** Martin Bialke, Dana Stahl, Torsten Leddig, Wolfgang Hoffmann

**Affiliations:** 1 Department Epidemiology of Health Care and Community Health, Institute for Community Medicine, University Medicine Greifswald, Ellernholzstr. 1-2, Greifswald, 17475, Germany, 49 3834 7580; 2 Trusted Third Party of the University Medicine Greifswald, University Medicine Greifswald, Greifswald, Germany

**Keywords:** architecture, scalability, trusted third party, application, security, consent, identifying data, infrastructure, modular, software, implementation, user interface, health platform, data management, data privacy, health record, electronic health record, EHR, pseudonymization

With great interest, we read the article *Development of a Trusted Third Party at a Large University Hospital: Design and Implementation Study* by Wündisch et al [1]. Its objective was to introduce a “comprehensive architecture for a TTP [trusted third party] that aims to support a wide range of different research projects,” incorporating “a fine-grained authentication and authorization model [and] a modern [REST-API (representational state transfer application programming interface)]” in order to “support cross-service workflows*.*” Their work is based on University Medicine Greifswald’s well-established software components for record linkage (E-PIX [Enterprise Identifier Cross-Referencing]), pseudonymization (gPAS [Generic Pseudonym Administration Service]), and consent management (gICS [Generic Informed Consent Service]) [2].

With this letter, we aim to place the authors’ statement—“the literature lacks insights into the design of more comprehensive architectures that support complex research workflows that are actually in production use”—into a state-of-the-art perspective to prevent any misleading impressions. While the authors concede that “research exists on the components mentioned above,” their article contains several inaccuracies that we would like to highlight.

The functional scope of the existing solutions (E-PIX, gPAS, gICS) is presented in Wündisch et al’s [1] first table. However, University Medicine Greifswald’s workflow management solution—TTP Dispatcher—is not displayed [2]. The authors only reference this highly relevant component later in their article.

Furthermore, the content and designation of Wündisch et al’s [1] second table—“additional functional requirements”—misleadingly suggests that the listed requirements are not covered by the solutions mentioned in their first table. In published work [2,3] and available materials [4], many of the check marks listed in the second table have been successfully validated, and the tools’ compliance with the pertinent Technology, Methods, and Infrastructure for Networked Medical Research guidelines [3] has been demonstrated.

Unlike the authors’ indication, University Medicine Greifswald’s TTP Dispatcher solution provides a common REST-API across all TTP services (based on E-PIX, gPAS, and gICS) and enables cross-service workflows [2]. Contrary to the description by Wündisch et al [1], the dispatcher architecture allows the implementation of complex research workflows. We published a list of available workflows together with a corresponding example (“automatic creation of pseudonyms linked to the primary identifier when registering a patient or study participant”) [2].

Since 2018, the existing TTP Dispatcher solution has been made available in various project collaborations [3]. In 2024, TTP Dispatcher is used in projects throughout Germany, and comprehensive documentation for the latest software version is publicly available [4].

Regarding the secure authentication mechanisms’ relevance, we fully agree with the authors that OAuth 2.0 support based on OpenID Connect and a fine-grained authorization model are essential for securing TTP services. Therefore, Keycloak support for E-PIX, gPAS, and gICS has been operational since 2022 [5].

We also encourage the authors’ interoperability endeavors with regard to HL7 FHIR (Health Level Seven International Fast Healthcare Interoperability Resources); University Medicine Greifswald has actively contributed to the HL7 FHIR standard and has fully implemented it [5].

We hope that our additions have clarified any remaining uncertainties and welcome further opportunities to exchange and share our practical experience with the authors.

## Abbreviations

E-PIX: Enterprise Identifier Cross-Referencing
gICS: Generic Informed Consent Service
gPAS: Generic Pseudonym Administration Service
HL7 FHIR: Health Level Seven International Fast Healthcare Interoperability Resources
REST-API: representational state transfer application programming interface
TTP: trusted third party
